# Associations Between Metal Levels in Whole Blood and IgE Concentrations in Pregnant Women Based on Data From the Japan Environment and Children’s Study

**DOI:** 10.2188/jea.JE20180098

**Published:** 2019-12-05

**Authors:** Mayumi Tsuji, Chihaya Koriyama, Yasuhiro Ishihara, Megumi Yamamoto, Kiwako Yamamoto-Hanada, Kumiko Kanatani, Yu Ait Bamai, Kazunari Onishi, Ayako Senju, Shunsuke Araki, Eiji Shibata, Seiichi Morokuma, Masafumi Sanefuji, Hiroshi Kitazawa, Mayako Saito, Masakazu Umezawa, Atsuto Onoda, Koichi Kusuhara, Rie Tanaka, Toshihiro Kawamoto, the Japan Environment & Children’s Study Group

**Affiliations:** 1 Department of Environmental Health, School of Medicine, University of Occupational and Environmental Health, Fukuoka, Japan; 2 Department of Epidemiology and Preventive Medicine, Kagoshima University Graduate School of Medical and Dental Sciences, Kagoshima, Japan; 3 Laboratory of Molecular Brain Science, Graduate School of Integrated Arts and Sciences, Hiroshima University, Hiroshima, Japan; 4 Department of Environment and Public Health, National Institute for Minamata Disease, Kumamoto, Japan; 5 Medical Support Center for Japan Environment and Children’s Study, National Center for Child Health and Development, Tokyo, Japan; 6 Department of Health Informatics, Graduate School of Medicine and Public Health, Kyoto University, Kyoto, Japan; 7 Department of Public Health, Hokkaido University Graduate School of Medicine, Sciences, Hokkaido, Japan; 8 Center for Birth Cohort Studies, Interdisciplinary Graduate School of Medicine, University of Yamanashi, Yamanashi, Japan; 9 Japan Environment and Children’s Study, UOEH Subunit Center, University of Occupational and Environmental Health, Fukuoka, Japan; 10 Department of Pediatrics, School of Medicine, University of Occupational and Environmental Health, Fukuoka, Japan; 11 Department of Obstetrics and Gynecology, School of Medicine, University of Occupational and Environmental Health, Fukuoka, Japan; 12 Research Center for Environmental and Developmental Medical Sciences, Kyushu University, Fukuoka, Japan; 13 Department of Materials Science and Technology, Faculty of Industrial Science and Technology, Tokyo University of Science, Tokyo, Japan; 14 Division of Neonatology, Center for Maternal-Neonatal Care, Nagoya University Hospital, Nagoya, Japan; 15 Postdoctoral Fellow of Japan Society for the Promotion of Science, Tokyo, Japan

**Keywords:** metal concentrations, specific IgE, pregnant women, maternal health, allergic sensitization

## Abstract

**Background:**

Metal exposures could possibly affect allergic responses in pregnant women, although no studies have yet shown a clear relationship between the two, and such exposures might also affect the development of allergic diseases in children.

**Methods:**

We investigated the relationship between metal concentrations in whole blood and immunoglobulin E (IgE; total and specific) in 14,408 pregnant women who participated in the Japan Environment and Children’s Study. The subjects submitted self-administered questionnaires, and blood samples were collected from them twice, specifically, during the first trimester and again during the second/third trimester. Concentrations of the metals Cd, Pb, Hg, Se, and Mn, as well as serum total and allergen-specific IgEs for egg white, house dust-mites (HDM), Japanese cedar pollen (JCP), animal dander, and moth, were measured. Allergen-specific IgE(s) were divided based on concentrations <0.35 or ≥0.35 UA/mL, and the metal levels were divided into quartiles.

**Results:**

Multivariable logistic regression analysis showed that there was a significant negative correlation between HDM- and animal dander-specific IgEs and Hg and Mn concentrations. Conversely, there was a significant positive relationship between JCP-specific IgE and Hg and Se concentrations.

**Conclusions:**

Metal exposures may be related to both increases and decreases in allergen-specific IgEs in pregnant women.

## INTRODUCTION

Allergic diseases can potentially affect the course of pregnancy,^1^ and poorly controlled allergic diseases during pregnancy are associated with high mortality, low birth weights, and congenital malformations.^2^ Maternal sensitization to allergens also affects the onset of allergic diseases in children.^3^^,^^4^ This is because the maternal allergy is associated with an increase of cord blood immunoglobulin E (IgE),^3^ which can potentially induce T helper (Th) 2 shifts in the neonate,^3^^,^^4^ and these Th2 shifts may contribute to the development of allergies in subsequent children.^4^

Currently, two types of IgE information are measured clinically in common allergy diagnoses.^5^^,^^6^ One type involves the measurement of total IgE, in which the antibody activity toward specific allergens is unclear. Total IgE concentrations are known to be associated with the development of allergic symptoms and sensitizations.^7^ The other type involves the measurement of specific IgEs, and these data show the antibody activity against specific allergens. Recently, the immunotoxic properties of metals have been pointed out and some studies have reported that there are relationships between metal exposures and IgEs. In human studies, positive associations between exposures to mercury (Hg)/lead (Pb) and serum levels of IgEs were reported.^8^^–^^10^ On the other hand, other studies have reported that there were relationships between Hg, cadmium (Cd), selenium (Se), and manganese (Mn) exposures and IgEs.^10^^–^^12^ However, the number of study subjects ranged from about only several tens of individuals to approximately one thousand, and there have been no studies that measured both total IgE and several specific IgEs at the same time.

Various changes occur in pregnant women compared to those who are not pregnant, and pregnant women tend to have higher metal concentrations in their bodies. For example, Cd absorption increases when iron stores are depleted.^13^ Therefore, the blood Cd concentration increases throughout the pregnancy period because pregnant women tend to exhaust iron stores.^13^ Additionally, bones are known reservoirs for Pb.^14^ Pregnancy and lactation are powerful stimuli for bone resorption, and Pb that reenters circulation can raise blood Pb levels in pregnant women.^14^^,^^15^ In this way, although the relationships between many metal concentrations and pregnancy have not been fully clarified, metals that women have been exposed to may have different in vivo metabolic fates and effects in pregnant women than those in women who are not pregnant.

Taken together, it is very important to survey maternal metal exposures and IgE levels for both maternal and childhood health. Therefore, we have tried to clarify the relationships between metal concentrations in blood and total and specific IgEs in pregnant women who participated in the Japan Environment and Children’s Study (JECS).^16^^,^^17^ This study is a cross-sectional study and the first of its kind in Japan to use JECS data to elucidate the connection between metal concentrations in blood and IgEs.

## MATERIALS AND METHODS

### Ethical approval and informed consent

JECS has been conducted based on the Ethical Guidelines for Epidemiological Research proposed by Japan’s Ministry of Health and Welfare (currently the Ministry of Health, Labour and Welfare). The JECS protocol was reviewed and approved by the Ministry of the Environment’s Institutional Review Board on Epidemiological Studies and by the Ethics Committees of all participating institutions. The JECS was conducted in accordance with the Helsinki Declaration and Japanese national regulations, with written informed consent obtained from all participants.^16^^,^^17^

### Subjects and questionnaires

The target subjects of this study were pregnant women (median age: 31 years) who participated in the JECS. They were recruited from January 2011 through March 2014. These women lived in one of 15 JECS study regions covering a wide geographical area of Japan, and they were recruited during early pregnancy at obstetric facilities and/or local government offices.^16^

**Figure 1.**
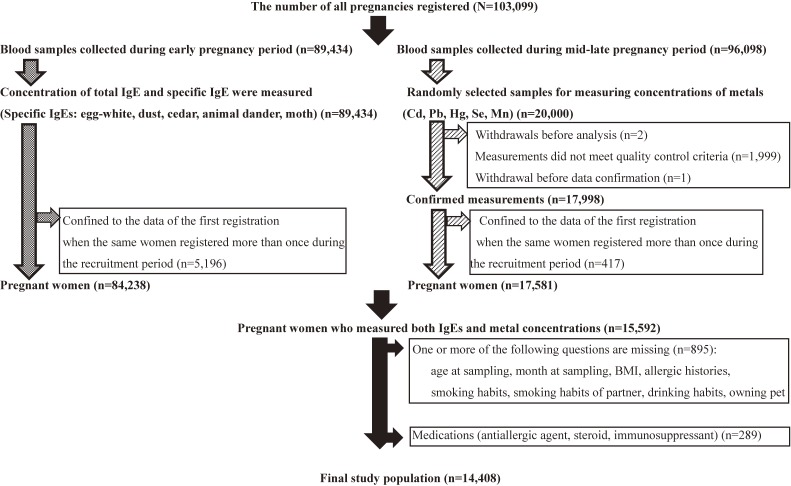
Flow chart showing the selection of the study subjects

Of the 15,592 women that had both IgEs and metal concentrations measured, we excluded those for whom data were missing (*n* = 895) and those on medication (*n* = 289). The final study population was 14,408 pregnant women (Figure 1).

Self-administered questionnaires were distributed to the women during their first trimester (T1) and second or third trimester (T2) at prenatal examinations or via mail. Data from the questionnaire given at T1 were used in this study because IgE levels were measured using T1 blood samples. Questionnaire data for the present study is based on the “jecs-ag-ai-20160424” dataset, which was released in June 2016 and revised in October 2016.

### Blood collection

Maternal blood samples were collected during T1 (median pregnancy week: 15) and T2 (median pregnancy week: 26) of pregnancy.^17^^,^^18^ In one round, 33 mL of blood were collected from each pregnant woman. Total IgE and allergen-specific IgE levels were then measure using T1 blood samples. Metal concentrations were measured using T2 blood samples. Blood sample data for the present study is based on the “jecs-mtl-ai-20170403” dataset, which was released in April 2017.

### Measurements of metal (Cd, Pb, Hg, Se, and Mn) concentrations in blood

The method used for measurements of metal concentrations in blood has already been published in our earlier paper.^19^ Briefly, blood samples were collected in tubes lined with sodium ethylenediaminetetraacetic acid (EDTA) and stored at −80°C until use. A standard solution of all target elements except Hg was prepared in 0.14 M nitric acid. A standard solution of Hg was produced in a mixture of 0.056 M nitric acid, 0.5% w/v H_4_EDTA, and 1% v/v tetramethylammonium hydroxide (TMAH). The final concentrations of Hg, Pb, Cd, Mn, and Se in the standard solutions were 200, 200, 20, 600, and 2,000 ng/g, respectively. Internal standards (yttrium, indium, and thallium, 250 ng/g) were prepared in 0.14 M nitric acid. Blood samples (200 µL) were diluted to a 1:19 ratio using a solution of 2% v/v butan-1-ol, 0.1% TMAH, 0.05% w/v polyoxyethylene (10) octylphenyl ether, and 0.05% w/v H_4_EDTA, and then, samples were vortex mixed prior to analysis with inductively coupled plasma mass spectrometry (ICP-MS).

Metal levels in the blood were measured using an Agilent 7700 inductively coupled plasma mass spectrometer (Agilent Technologies, Japan). Method detection limits for each analyte were calculated as described previously.^20^ The total concentration of the Pb isotypes is given as the sum of the concentrations of ^206^Pb, ^207^Pb, and ^208^Pb. To correct the spectral overlap from molybdenum oxide (^95^Mo^16^O), the concentration of ^111^Cd was calculated using the following equation: [Cd] = [m/z 111 concentration] − [m/z 95 concentration] × [^95^Mo^16^O generation rate]. The ^95^Mo^16^O generation rate was derived using the equation [^95^Mo^16^O generation rate] = [concentration of m/z 111 when a standard solution of 100 ppb ^95^Mo was analyzed]/[concentration of m/z 96 when a standard solution of 100 ppb ^95^Mo was analyzed].

### Total and allergen-specific IgE assays

Serum total and allergen-specific IgE levels were analyzed using immunological assays with the ImmunoCAP system (Thermo Fisher Scientific, Sweden) at a clinical laboratory under contract. The following specific allergens were detected: egg white, house dust-mite (HDM; *Dermatophagoides pteronyssinus*), Japanese cedar pollen (JCP), animal dander, and moth. The results of the ImmunoCAP system were given as exact values in UA/mL, and these data were scored using the following system of seven classes ranging from 0–6: class 0: <0.35, class 1: 0.35–0.69, class 2: 0.70–3.49, class 3: 3.50–17.49, class 4: 17.50–49.99, class 5: 50.00–99.99, and class 6: ≥100. In the results, values of 0.35 UA/mL or higher were taken as a sign of sensitization.^21^

### Statistical analyses

The distribution of metals was skewed; therefore, natural logarithmic conversions were applied to the variables before two-group comparisons using Welch’s *t*-test and multivariable regression analysis. The associations between specific IgE classes and metal concentrations were analyzed using multivariable regression. Adjusted means, 95% confidence intervals, and *P* values were calculated using multivariable logistic regression and multivariable regression analysis with adjusted factors, such as age, body mass index (BMI) before pregnancy, allergic diseases (asthma, allergic rhinitis, atopic dermatitis, allergic conjunctivitis, food allergy, drug allergy), smoking habits, partner’s smoking habits, alcohol consumption, ownership of pets, the month of T1 blood sampling, and geographic region.

Participants were asked to report allergic diseases diagnosed by clinicians. For the category of smoking habits, current smokers and never-/former-smokers were categorized as “Yes” and “No,” respectively. For alcohol consumption, women who consumed alcohol during pregnancy were grouped as “Yes” and women who had never consumed or quit drinking during pregnancy were grouped as “No.” We treated smoking and alcohol consumption as categorical variables in the statistical models.

We divided all subjects into four groups depending on the concentration levels of each metal, so that each sub-group consisted of close to one-fourth of the total subjects (ie, a “quartile”; 1^st^ quartile = Q1, 2^nd^ quartile = Q2, 3^rd^ quartile = Q3, 4^th^ quartile = Q4), and we used these sub-groups in the multivariable logistic regression analysis.

All analyses were performed in STATA version 14 (Stata Corporation, College Station, TX, USA), and statistical significance was assumed when *P* < 0.05 (two-sided).

## RESULTS

Table 1 shows the characteristics of the study population, and Table 2 shows the total and allergen-specific IgE classes in serum. No subject had an egg-specific IgE class of 4 or higher or a moth-specific IgE class of 5 or more. On the other hand, the subjects had HDM-, JCP-, and animal dander-specific IgE results that were distributed across all of the classes.

**Table 1.** Characteristics of the study population

Characteristics	
**Mean (SD) age, years**	30.9 (4.9)
**Mean (SD) BMI, kg/m^2^**	21.2 (3.2)
**Asthma**	
No/Yes (%)	12,890 (89.5)/1,518 (10.5)
**Allergic rhinitis**	
No/Yes (%)	9,359 (65.0)/5,049 (35.0)
**Atopic dermatitis**	
No/Yes (%)	12,134 (84.2)/2,274 (15.8)
**Allergic conjunctivitis**	
No/Yes (%)	12,988 (90.1)/1,420 (9.9)
**Food allergy**	
No/Yes (%)	13,750 (95.4)/658 (4.6)
**Drug allergy**	
No/Yes (%)	14,048 (97.5)/360 (2.5)
**Smoking during pregnancy**	
No/Yes (%)	13,723 (95.2)/685 (4.8)
**Smoking during pregnancy (partner)**	
No/Yes (%)	7,847 (54.5)/6,561 (45.5)
**Alcohol consumption during pregnancy**	
No/Yes (%)	12,959 (89.9)/1,449 (10.1)
**Own pets**	
No/Yes (%)	11,214 (77.8)/3,194 (22.2)
**Total IgE concentration, IU/mL**	
≤173/>173 (%)	11,133 (77.3)/3,275 (22.7)
**Mean (SD) metal concentration, ng/g**	
Cd	0.75 (0.38)
Pb	6.44 (2.86)
Hg	4.17 (2.41)
Se	171 (20.4)
Mn	15.8 (4.61)

BMI, body mass index; IgE, immunoglobulin E; SD, standard deviation.

**Table 2.** Serum allergen-specific IgE data for the study population

	Number of the subjects (%)						

Specific IgE concentration class^a^	0	1	2	3	4	5	6
Egg white	14,260 (99.0)	101 (0.7)	45 (0.3)	2 (0.01)	0 (0)	0 (0)	0 (0)
HDM	7,606 (52.8)	809 (5.6)	1,639 (11.4)	2,356 (16.4)	1,352 (9.4)	474 (3.3)	172 (1.2)
JCP	6,453 (44.8)	708 (4.9)	1,984 (13.8)	2,900 (20.1)	1,629 (11.3)	549 (3.8)	185 (1.3)
Animal dander	11,438 (79.4)	948 (6.6)	1,413 (9.8)	454 (3.2)	118 (0.8)	31 (0.2)	6 (0.04)
Moth	10,331 (71.7)	1,345 (9.3)	2,146 (14.9)	557 (3.9)	29 (0.2)	0 (0)	0 (0)

IgE, immunoglobulin E.

Values are given in numbers and percentages.

^a^class 0: <0.35, class 1: 0.35–0.69, class 2: 0.70–3.49, class 3: 3.5–17.49, class 4: 17.5–49.99, class 5: 50–99.99, class 6: ≥100 (UA/mL).

The concentration of Cd in the samples from those who had the low egg white-specific IgE was significantly higher than that from subjects who had high IgE values for that allergen. A similar tendency was also observed for HDM-specific IgE and Pb, as well as animal dander-specific IgE and Cd, Hg, and Se. However, the Hg and Se concentrations in samples with high JCP-specific IgE values were significantly higher than those in samples with low IgE values for the same allergen (Table 3).

**Table 3.** The relationships between metal concentrations and total and allergen-specific IgEs

Metal concentration median (25th, 75th)	Total IgE, IU/mL			Egg white-specific IgE, UA/mL			HDM-specific IgE, UA/mL		
		
	Low (≤173; *n* = 11,133)	High (>173; *n* = 3,275)	*P* value^a^	Low (<0.35; *n* = 14,260)	High (≥0.35; *n* = 148)	*P* value^a^	Low (<0.35; *n* = 7,606)	High (≥0.35; *n* = 6,802)	*P* value^a^
Cd	0.66 (0.50, 0.90)	0.65 (0.49, 0.88)	0.059	0.66 (0.50, 0.90)	0.60 (0.47, 0.84)	0.018	0.66 (0.50, 0.90)	0.65 (0.49, 0.90)	0.454
Pb	5.91 (4.78, 7.40)	5.99 (4.81, 7.49)	0.402	5.93 (4.79, 7.43)	5.62 (4.63, 7.35)	0.274	5.98 (4.80, 7.46)	5.88 (4.77, 7.39)	0.016
Hg	3.63 (2.57, 5.13)	3.58 (2.53, 5.12)	0.732	3.62 (2.56, 5.12)	3.64 (2.43, 5.41)	0.971	3.63 (2.58, 5.19)	3.61 (2.54, 5.09)	0.240
Se	169 (157, 182)	169 (157, 183)	0.526	169 (157, 182)	170 (157, 188)	0.389	169 (158, 183)	169 (157, 182)	0.045
Mn	15.2 (12.5, 18.5)	15.2 (12.6, 18.4)	0.952	15.2 (12.5, 18.5)	16.0 (12.7, 19.1)	0.320	15.2 (12.5, 18.5)	15.2 (12.6, 18.5)	0.683

Metal concentration median (25th, 75th)	JCP-specific IgE, UA/mL			Animal dander-specific IgE, UA/mL			Moth-specific IgE, UA/mL		
		
	Low (<0.35; *n* = 6,453)	High (≥0.35; *n* = 7.955)	*P* value^a^	Low (<0.35; *n* = 11,438)	High (≥0.35; *n* = 2,970)	*P* value^a^	Low (<0.35; *n* = 10,331)	High (≥0.35; *n* = 4,077)	*P* value^a^
Cd	0.66 (0.50, 0.91)	0.66 (0.49, 0.89)	0.308	0.66 (0.50, 0.90)	0.64 (0.48, 0.88)	0.003	0.66 (0.49, 0.90)	0.66 (0.50, 0.90)	0.574
Pb	5.92 (4.77, 7.43)	5.94 (4.79, 7.43)	0.837	5.93 (4.78, 7.43)	5.93 (4.79, 7.42)	0.594	5.90 (4.77, 7.41)	6.00 (4.81, 7.46)	0.105
Hg	3.48 (2.47, 4.91)	3.76 (2.64, 5.28)	<0.001	3.65 (2.58, 5.16)	3.52 (2.50, 5.02)	0.006	3.64 (2.58, 5.13)	3.58 (2.50, 5.12)	0.237
Se	168 (156, 182)	170 (158, 183)	<0.001	169 (157, 183)	168 (157, 182)	0.024	169 (157, 183)	169 (157, 182)	0.574
Mn	15.2 (12.5, 18.5)	15.2 (12.5, 18.5)	0.797	15.2 (12.6, 18.5)	15.1 (12.3, 18.5)	0.056	15.2 (12.6, 18.5)	15.1 (12.5, 18.4)	0.097

IgE, immunoglobulin E.

^a^*P* values were obtained using Welch’s *t*-test with natural log transformed values.

To further assess the association between metal concentrations and IgE levels, while considering adjusted factors that could have influences on allergic sensitizations, we conducted multivariable logistic regression analyses using the quartile variables of the metal concentrations. The allergen-specific IgEs were divided into the following two groups: low: <0.35 or high: ≥0.35 (UA/mL). There were significant relationships between HDM-specific IgE and Hg Q4; JCP-specific IgE and Hg Q3 and Q4; JCP-specific IgE and Se Q2, Q3, and Q4; animal dander-specific IgE and Hg Q3 and Q4; animal dander-specific IgE and Se Q3; and animal dander-specific IgE and Mn Q2 and Q3. The odds ratio (OR) of HDM- and animal dander-specific IgE decreased as the Hg concentration increased (*P* values for the trends were 0.010 and 0.006, respectively). Conversely, the OR of JCP-specific IgE increased as the Hg and Se concentrations increased (*P* values for the trends were both <0.001) (Table 4). There were significant relationships between total IgE values and Se Q4; and moth-specific IgE and Cd Q3. However, the ORs of total IgE and moth-specific IgE did not increase or decrease significantly as the Se and Cd concentrations changed (*P* values for the trends were 0.842 and 0.206, respectively) (eTable 1).

**Table 4.** Results of multivariable analysis in the relationship between quartile concentration of metals and allergen-specific IgEs

Quartile concentration of metals (ng/g)	HDM-specific IgE, UA/mL				JCP-specific IgE, UA/mL				Animal dander-specific IgE, UA/mL			
		
	Low (<0.35; *n* = 7,606)	High (≥0.35; *n* = 6,802)	OR (95% CI)^a^	*P* value^a^	Low (<0.35; *n* = 6,453)	High (≥0.35; *n* = 7,955)	OR (95% CI)^a^	*P* value^a^	Low (<0.35; *n* = 11,438)	High (≥0.35; *n* = 2,970)	OR (95% CI)^a^	*P* value^a^
**Cd**												
Q1 (≤0.495)	1,841	1,754	1.00 (referent)		1,586	2,009	1.00 (referent)		2,797	798	1.00 (referent)	
Q2 (0.496–0.657)	1,924	1,674	0.92 (0.84–1.02)	0.100	1,628	1,970	0.94 (0.85–1.04)	0.228	2,831	767	0.98 (0.87–1.10)	0.746
Q3 (0.658–0.897)	1,943	1,661	0.94 (0.85–1.04)	0.225	1,570	2,034	1.04 (0.94–1.15)	0.464	2,905	699	0.90 (0.80–1.02)	0.097
Q4 (≥0.898)	1,898	1,713	1.04 (0.94–1.15)	0.498	1,669	1,942	0.98 (0.89–1.09)	0.747	2,905	706	0.94 (0.83–1.07)	0.368
**Pb**												
Q1 (≤4.78)	1,866	1,734	1.00 (referent)		1,623	1,977	1.00 (referent)		2,860	740	1.00 (referent)	
Q2 (4.79–5.92)	1,855	1,736	1.00 (0.90–1.10)	0.935	1,618	1,973	1.00 (0.91–1.11)	0.953	2,850	741	0.99 (0.87–1.11)	0.815
Q3 (5.93–7.42)	1,937	1,670	0.92 (0.84–1.02)	0.103	1,591	2,016	1.06 (0.96–1.17)	0.252	2,858	749	1.01 (0.90–1.14)	0.870
Q4 (≥7.43)	1,948	1,662	0.91 (0.83–1.01)	0.073	1,621	1,989	1.04 (0.94–1.15)	0.407	2,870	740	0.99 (0.88–1.12)	0.885
**Hg**												
Q1 (≤2.55)	1,858	1,726	1.00 (referent)		1,754	1,830	1.00 (referent)		2,801	783	1.00 (referent)	
Q2 (2.56–3.61)	1,926	1,680	0.91 (0.82–1.00)	0.057	1,684	1,922	1.06 (0.96–1.18)	0.214	2,845	761	0.93 (0.83–1.05)	0.238
Q3 (3.62–5.11)	1,856	1,727	0.98 (0.89–1.08)	0.734	1,543	2,040	1.23 (1.12–1.36)	<0.001	2,865	718	0.88 (0.78–0.99)	0.031
Q4 (≥5.12)	1,966	1,669	0.86 (0.78–0.95)	0.003	1,472	2,163	1.35 (1.22–1.49)	<0.001	2,927	708	0.81 (0.72–0.92)	0.001
**Se**												
Q1 (≤156)	1,755	1,659	1.00 (referent)		1,624	1,790	1.00 (referent)		2,680	734	1.00 (referent)	
Q2 (157–168)	1,878	1,719	1.00 (0.90–1.10)	0.951	1,617	1,980	1.15 (1.04–1.27)	0.008	2,813	784	1.06 (0.94–1.19)	0.355
Q3 (169–181)	1,948	1,663	0.91 (0.82–1.01)	0.063	1,593	2,018	1.17 (1.06–1.30)	0.002	2,912	699	0.88 (0.78–1.00)	0.046
Q4 (≥182)	2,025	1,761	0.94 (0.85–1.04)	0.233	1,619	2,167	1.26 (1.14–1.39)	<0.001	3,033	753	0.95 (0.84–1.07)	0.414
**Mn**												
Q1 (≤12.4)	1,856	1,628	1.00 (referent)		1,550	1,934	1.00 (referent)		2,711	773	1.00 (referent)	
Q2 (12.5–15.1)	1,924	1,704	1.01 (0.92–1.12)	0.793	1,666	1,962	0.97 (0.87–1.07)	0.494	2,900	728	0.87 (0.77–0.98)	0.024
Q3 (15.2–18.4)	1,907	1,754	1.03 (0.94–1.14)	0.519	1,614	2,047	1.03 (0.93–1.14)	0.594	2,941	720	0.83 (0.74–0.94)	0.002
Q4 (≥18.5)	1,919	1,716	1.01 (0.92–1.12)	0.794	1,623	2,012	1.02 (0.92–1.13)	0.681	2,886	749	0.90 (0.79–1.01)	0.070

IgE, immunoglobulin E.

^a^Odds ratios and corresponding 95% confidence intervals and *P* values were obtained using multivariable logistic regression analysis adjusted for age, BMI, allergic diseases (asthma, allergic rhinitis, atopic dermatitis, allergic conjunctivitis, food allergy, drug allergy), smoking during pregnancy, smoking habits of partner, alcohol consumption during pregnancy, owning pets, month of T1 blood sampling, and geographic region.

We analyzed the relationships between allergen-specific IgE classes and metal concentrations that had significant differences (Table 4). Hg concentrations decreased significantly depending on the class of HDM-specific IgE (*P* = 0.002). Hg and Mn concentrations decreased significantly depending on the classes of animal dander-specific IgE (Hg: *P* < 0.001; Mn: *P* = 0.033). On the other hand, Hg and Se concentrations increased significantly depending on the classes of JCP-specific IgE (Hg: *P* < 0.001; Se: *P* < 0.001) (Table 5).

**Table 5.** The relationship between metal concentrations and allergen-specific IgE scores

	Adjusted^b^ mean (95% CI) of the metal concentration, ng/g							
Antigen-specific IgE classes^a^	0	1	2	3	4	5	6	*P* for trend^c^
**HDM**								

Hg	3.67 (3.63, 3.71)	3.64 (3.61, 3.67)	3.61 (3.57, 3.64)	3.58 (3.53, 3.62)	3.55 (3.49, 3.61)	3.52 (3.44, 3.59)	3.49 (3.39, 3.58)	0.002
**JCP**								

Hg	3.49 (3.45, 3.54)	3.57 (3.54, 3.61)	3.65 (3.62, 3.69)	3.74 (3.70, 3.78)	3.82 (3.76, 3.88)	3.91 (3.83, 3.99)	4.00 (3.90, 4.10)	<0.001
Se	169 (168, 169)	169 (169, 170)	170 (170, 170)	171 (170, 171)	171 (171, 172)	172 (171, 173)	173 (172, 174)	<0.001
**Animal dander**								

Hg	3.66 (3.63, 3.70)	3.58 (3.54, 3.61)	3.49 (3.43, 3.56)	3.41 (3.32, 3.51)	3.33 (3.21, 3.46)	3.25 (3.10, 3.41)	3.18 (3.00, 3.37)	<0.001
Mn	15.2 (15.1, 15.3)	15.1 (15.0, 15.2)	15.0 (14.9, 15.2)	14.9 (14.7, 15.2)	14.9 (14.6, 15.2)	14.8 (14.4, 15.2)	14.7 (14.2, 15.1)	0.033

CI, confidence interval; HDM, house dust mites; JCP, Japanese cedar pollen.

^a^class 0: <0.35, class 1: 0.35–0.69, class 2: 0.70–3.49, class 3: 3.5–17.49, class 4: 17.5–49.99, class 5: 50–99.99, class 6: ≥100 (UA/mL).

^b^Age, BMI, allergic diseases (asthma, allergic rhinitis, atopic dermatitis, allergic conjunctivitis, food allergy, drug allergy), smoking during pregnancy, smoking habits of partner, alcohol consumption during pregnancy, owning pets, month of T1 blood sampling, and geographic region were adjusted for.

^c^*P* for trend was obtained using multivariable regression analysis with natural log transformed metal concentrations.

## DISCUSSION

This study demonstrated that there is a positive relationship between JCP-specific IgE and Hg and Se concentrations in the blood of pregnant women. However, HDM- and animal dander-specific IgE had a negative relationship with Hg concentrations.

Hg and Se are both taken in mainly from seafood, and since there is a high interest in Hg intake in Japan, the Japanese government has announced precautions regarding the consumption of seafood during pregnancy. There are only a few epidemiological studies that have reported on the association between Hg and IgEs in humans. Grandjean et al reported that prenatal methylmercury (MeHg) exposures were slightly associated with an increase in total IgE; however, grass-specific IgE was inversely associated with prenatal MeHg exposures in children.^11^ Weidinger et al reported a positive relation between Hg and total IgE but not specific IgE in children.^8^ In a study using human peripheral blood mononuclear cells to investigate the effects of MeHg and inorganic Hg (InHg) on immunologic dysfunction, interleukin 4 (IL-4) increased in association with both MeHg and InHg exposures, and in particular, the increases in concentrations of IL-4 were lower for MeHg than those for InHg.^22^ In studies using rats and mice, InHg was found to induce the activation of Th2 cells and the upregulation of IL-4, and it activated polyclonal B cells and increased the level of IgE in circulation.^23^^–^^25^ Although the different mechanisms of the influence of Hg exposures on total IgE and specific IgE are not known, considering human and animal studies together, increased IgE may be due to the secretion of IL-4 from Th2 cells activated by Hg exposures.

In an investigation of the natural history of the allergens, an analysis of the distribution of sensitization over time revealed that JCP-specific IgE reaches a peak in humans at the age of 40 years and then decreases rapidly thereafter.^26^ Since the average age of the participants in this study was 31 years, and 97% of the subjects were under 40 years of age, it is possible that current Hg exposures might affect the Th2 responses and increased the JCP-specific IgE in this study. In addition, this allergen is only present during a specific season. A strong increase in pollen-specific IgE was observed during the pollen season, and although it decreased once the season ended, it remained elevated for 1 year.^27^ Therefore, the relationship between JCP-specific IgE and Hg exposures could be explained by the direct promotion of Th2 by Hg exposures during the JCP pollen season, but it is necessary to consider other mechanisms during the non-JCP-pollen season. It has been reported that information is relayed to the immune system by IgE-switched B cells, which are not responsive to IL-4-mediated signals.^27^ Previous studies have shown that B cell receptor (BCR) signaling can be activated by spleen tyrosine kinase (Syk) via phosphorylation reactions in B cells.^28^ And Vaillant et al indicated that the BCR signaling pathway was also related with an increase in antibody production.^29^ When JCP enters the human body as an allergen, IgE-switched B cells may not die because of the abnormal BCR signaling pathway, and this may promote the formation of memory B cells and long-lived plasma cells, which can induce excessive IgE production resulting in an allergic response.^30^ McCabe et al^31^ reported that low levels of InHg attenuate the BCR signaling pathway, and Caruso et al^32^ reported that Hg alters the BCR signaling pathway, especially through inducing changes in Syk activity in experimental studies. In short, abnormalities in the BCR signaling pathway may occur in subjects who have been exposed to Hg, and consequently, IgE-switched B cells do not die and JCP-specific IgE is more likely to be produced throughout the year.

Deficiencies of Se, which is an essential element, generally pose a risk for negative effects on the immune systems and brains of pregnant women and fetuses.^33^ However, Se is toxic to humans when taken in at high doses through acute inhalation or dermal contact.^34^^–^^36^ Hoffmann^37^ reported that the relationship between Se and Th2 responses is not dose-dependent. One possible effect of Se is the induction of the Th1 immune system according to the mouse model^38^; however, this relationship is not consistent with the results in humans. Therefore, further studies are necessary to clarify the relationship between Se exposures and allergic responses.

The HDM-specific IgE had a negative relationship with Hg, and animal dander-specific IgE had a negative relationship with Hg and Mn. The mechanism of IgE sensitization to aeroallergens can change depending on the patient’s age.^39^ Unlike JCP, HDM and animal dander sensitizations are prominent in children,^40^ and HDM tends to not become a newly sensitized antigen as a person grows to adulthood.^39^^,^^40^ In addition, once HDM-specific IgE responses increase in childhood, it is difficult for decreases to occur.^41^ Therefore, adulthood HDM and animal dander-specific IgEs do not necessarily indicate accurately sensitizations in adulthood and it was difficult to draw a clear conclusion from the results of this study. In order to clarify the relationship between HDM- and animal dander-specific IgEs and metal exposures, further studies that include metal exposures and the allergic sensitization situation during childhood will be necessary.

**Figure 2.**
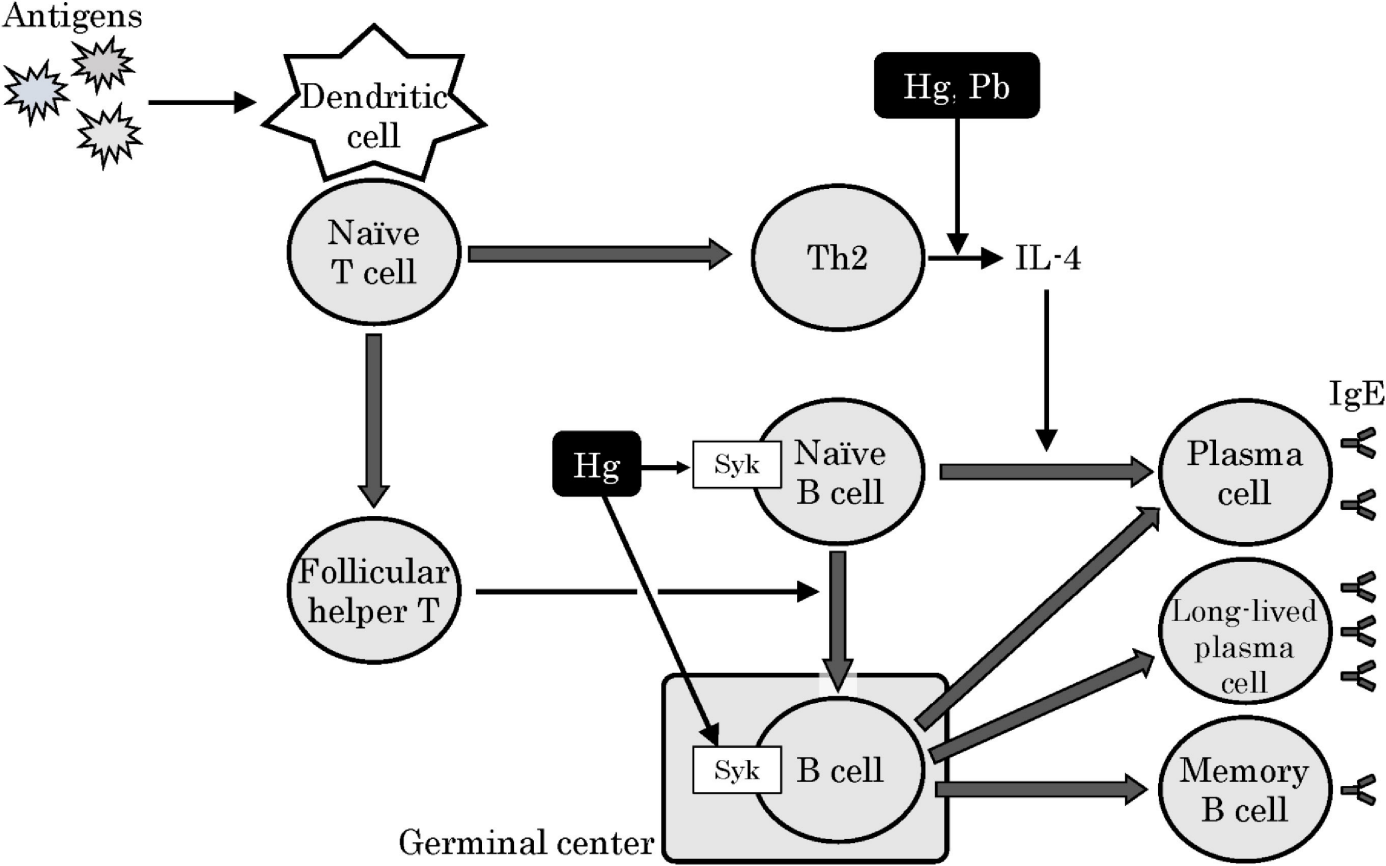
Hypothetical mechanistic scheme for the modulation of IgE production by heavy metals. There are mainly two pathways that are involved in the production of IgE; these include a Th2-derived IL-4 dependent pathway and T follicular helper (Tfh) cell-dependent, IL-4-independent pathway. Our results and previous work indicate that Pb or Hg exposures can induce several types of IgE. Additionally, these exposures can reportedly stimulate IL-4 production, thus suggesting that heavy metal-dependent increases in IL-4 levels might be one of the causes of IgE production. Also, Hg exposures are known to alter the phosphorylation status of Syk (spleen tyrosine kinase), which mediates B cell differentiation. This could be an alternative for the induction of IgE production, especially by Hg.

Based on our discussion and the results of previous research, we offer the following hypothesis on the mechanism for the upregulation of IgE production by heavy metals, which is related to allergic disease (Figure 2). There are mainly two pathways that produce IgE, specifically, pathways that are responsive or non-responsive to IL-4.^30^^,^^42^^,^^43^ Several researchers have reported that IL-4 expression is induced in the presence of Pb and Hg in both in vivo and in vitro studies.^22^^–^^24^^,^^44^^,^^45^ Hg exposures were also reported to alter the phosphorylation status of Syk in the BCR signaling pathway associated with IL-4-independent IgE production in WEHI-231 cells, a murine immature B lymphoma cell line.^31^^,^^32^ Taken together, Hg may interact with some components involved in the pathway of IgE production, and this is followed by an increase of IgE.

The present study has some limitations that merit discussion and further research. First, our target subject was only pregnant women. Pregnancy hormones, such as progesterone and human chorionic gonadotrophin, promote Th2 immunity.^46^ There is a notable study that showed that IL-4 can be produced under the influence of progesterone.^47^ Therefore, it is impossible to deny the possibility that pregnancy itself affects IgE production, and that the relationship between metal exposures and IgE might show stronger results in pregnant women compared to non-pregnant women. Regardless, a study targeting non-pregnant women is also needed in the future. Second, we could not determine the source of the exposure to the metals. According to the Japanese Ministry of Health, Labor and Welfare, however, more than 80% of Hg intake, which was significant in the present study, is derived from seafood.^48^ However, an investigation of whether occupational exposures are a factor is also needed. Third, because of the limited volume of blood collected from each pregnant woman, IgE and metal concentrations were measured using blood specimens collected at different times. The half-life of Hg in fish-eating humans is a few months.^49^ In the case of continuous exposure, a single-compartment model with a 70-day half-time predicts that the whole-body steady state (where intake equals excretion).^49^ In the present study, the food frequency questionnaire survey was conducted twice, in T1 and T2, and only 1% of the participants changed their fish consumption between the two surveys. Thus, we assumed that Hg exposure levels used in the present analysis reflect the exposure levels in T1, when blood IgEs were measured. The half-life of other metals is about 10 years for Cd, 5 years for Pb, a few months for Se, and a month for Mn.^50^^,^^51^ In particular, Mn has a short half-life and the fact that the periods for Mn and IgE measurements were different might have influenced our results. Further investigations using a proper study design for such metals are warranted to confirm our findings. Fourth, Cd, Hg, and Mn concentrations in our samples were higher than those in other countries, while Se and Pb levels were similar.^52^^–^^54^ Therefore, it is unknown whether similar results would be obtained in other countries, and additional research is warranted.

### Conclusion

This is the first study to link metal concentrations with IgEs in pregnant Japanese women. We showed that metal exposures may be related to both increases and decreases in allergen-specific IgEs and that JCP-specific IgE, in particular, was positively related to Hg concentrations.

## APPENDIX A. SUPPLEMENTARY DATA

eTable 1. Results of the multivariable analysis for the relationship between the quartile concentration of metals and total and allergen-specific IgEs
