# Supplementary material for: Associations Between Metal Levels in Whole Blood and IgE Concentrations in Pregnant Women Based on Data From the Japan Environment and Children’s Study
Source: J Epidemiol. 2019 Dec 5;29(12):478–86. doi: 10.2188/jea.JE20180098 (PMC6859078; doi:10.2188/jea.JE20180098)
Supplement: Supplementary file 1 [file je-29-478-s001.pdf]

eTable 1. Results of the multivariable analysis for the relationship between the quartile concentration of metals and total and allergen-specific IgEs

	**Total IgE, IU/mL**				**Egg white-specific IgE, UA/mL**				**Moth-specific IgE, UA/mL**			
Quartile concentration of metals, ng/g	Low (<0.35; n=11,133)	High (≥0.35; n=3,275)	OR (95% CI)[a]	P value[a]	Low (<0.35; n=14,260)	High (≥0.35; n=148)	OR (95% CI)[a]	P value[a]	Low (<0.35; n=10,331)	High (≥0.35; n=4,077)	OR (95% CI)[a]	P value[a]
**Cd**												
Q1 (≤0.495)	2,752	843	1.00 (referent)		3,547	48	1.00 (referent)		2,598	997	1.00 (referent)	
Q2 (0.496–0.657)	2,748	850	1.04 (0.93–1.16)	0.516	3,562	36	0.79 (0.51–1.23)	0.298	2,582	1,016	1.06 (0.95–1.17)	0.297
Q3 (0.658–0.897)	2,800	804	0.99 (0.88–1.11)	0.872	3,568	36	0.83 (0.53–1.30)	0.424	2,564	1,040	1.12 (1.00–1.24)	0.042
Q4 (≥0.898)	2,833	778	0.96 (0.85–1.08)	0.519	3,583	28	0.71 (0.43–1.15)	0.163	2,587	1,024	1.09 (0.98–1.22)	0.113
**Pb**												
Q1 (≤4.78)	2,802	798	1.00 (referent)		3,559	41	1.00 (referent)		2,612	988	1.00 (referent)	
Q2 (4.79–5.92)	2,800	791	0.98 (0.87–1.10)	0.704	3,548	43	1.05 (0.68–1.62)	0.822	2,604	987	0.98 (0.88–1.09)	0.730
Q3 (5.93–	2,772	835	1.05	0.362	3,577	30	0.73	0.206	2,546	1,061	1.07	0.182

7.42)			(0.94–1.18)				(0.46–1.18)				(0.97–1.19)	
Q4 (≥7.43)	2,759	851	1.08 (0.96–1.21)	0.213	3,576	34	0.85 (0.53–1.36)	0.498	2,569	1,041	1.02 (0.92–1.13)	0.691
**Hg**												
Q1 (≤2.55)	2,749	835	1.00 (referent)		3,542	42	1.00 (referent)		2,527	1,057	1.00 (referent)	
Q2 (2.56–3.61)	2,783	823	0.96 (0.85–1.07)	0.433	3,575	31	0.71 (0.45–1.14)	0.160	2,595	1,011	0.93 (0.84–1.03)	0.159
Q3 (3.62–5.11)	2,790	793	0.92 (0.82–1.03)	0.167	3,552	31	0.72 (0.45–1.16)	0.175	2,596	987	0.91 (0.82–1.01)	0.067
Q4 (≥5.12)	2,811	824	0.92 (0.82–1.04)	0.171	3,591	44	0.98 (0.63–1.50)	0.908	2,613	1,022	0.93 (0.84–1.03)	0.183
**Se**												
Q1 (≤156)	2,657	757	1.00 (referent)		3,383	31	1.00 (referent)		2,439	975	1.00 (referent)	
Q2 (157–168)	2,758	839	1.10 (0.98–1.23)	0.114	3,556	41	1.29 (0.80–2.06)	0.295	2,547	1,050	1.03 (0.93–1.15)	0.556
Q3 (169–181)	2,825	786	0.99 (0.88–1.11)	0.817	3,577	34	1.05 (0.64–1.72)	0.844	2,621	990	0.94 (0.85–1.05)	0.256
Q4 (≥182)	2,893	893	1.12 (1.00–1.26)	0.046	3,744	42	1.28 (0.80–2.05)	0.308	2,724	1,062	0.97 (0.87–1.07)	0.534
**Mn**												
Q1 (≤12.4)	2,702	782	1.00		3,451	33	1.00		2,473	1,911	1.00	

			(referent)				(referent)				(referent)	
Q2 (12.5–15.1)	2,791	837	1.04 (0.93–1.17)	0.484	3,597	31	0.90 (0.55–1.47)	0.673	2,597	1,031	0.97 (0.88–1.08)	0.610
Q3 (15.2–18.4)	2,821	840	1.02 (0.91–1.14)	0.736	3,620	41	1.16 (0.73–1.84)	0.542	2,643	1,018	0.94 (0.85–1.04)	0.227
Q4 (≥18.5)	2,819	816	1.00 (0.89–1.12)	0.992	3,592	43	1.23 (0.77–1.95)	0.382	2,618	1,017	0.96 (0.86–1.06)	0.396

[a]Odds ratios and corresponding 95% confidence intervals and P values were obtained using multivariable logistic regression analysis adjusted for age, BMI, allergic diseases (asthma, allergic rhinitis, atopic dermatitis, allergic conjunctivitis, food allergy, drug allergy), smoking during pregnancy, smoking habits of partner, alcohol consumption during pregnancy, owning pets, month of T1 blood sampling, and geographic region.
